# Genetic Determinants Associated With *in Vivo* Survival of *Burkholderia cenocepacia* in the *Caenorhabditis elegans* Model

**DOI:** 10.3389/fmicb.2018.01118

**Published:** 2018-05-29

**Authors:** Yee-Chin Wong, Moataz Abd El Ghany, Raeece N. M. Ghazzali, Soon-Joo Yap, Chee-Choong Hoh, Arnab Pain, Sheila Nathan

**Affiliations:** ^1^School of Biosciences and Biotechnology, Faculty of Science and Technology, Universiti Kebangsaan Malaysia, Bangi, Malaysia; ^2^Biological and Environmental Sciences and Engineering Division, King Abdullah University of Science and Technology, Thuwal, Saudi Arabia; ^3^The Westmead Institute for Medical Research and The Marie Bashir Institute for Infectious Diseases and Biosecurity, The University of Sydney, Sydney, NSW, Australia; ^4^Codon Genomics, Seri Kembangan, Malaysia

**Keywords:** *Burkholderia cenocepacia*, *Caenorhabditis elegans*, essential genes, TraDIS, *in vivo* survival

## Abstract

A *Burkholderia cenocepacia* infection usually leads to reduced survival and fatal *cepacia* syndrome in cystic fibrosis patients. The identification of *B. cenocepacia* essential genes for *in vivo* survival is key to designing new anti-infectives therapies. We used the Transposon-Directed Insertion Sequencing (TraDIS) approach to identify genes required for *B. cenocepacia* survival in the model infection host, *Caenorhabditis elegans*. A *B. cenocepacia* J2315 transposon pool of ∼500,000 mutants was used to infect *C. elegans*. We identified 178 genes as crucial for *B. cenocepacia* survival in the infected nematode. The majority of these genes code for proteins of unknown function, many of which are encoded by the genomic island BcenGI13, while other gene products are involved in nutrient acquisition, general stress responses and LPS *O*-antigen biosynthesis. Deletion of the glycosyltransferase gene *wbxB* and a histone-like nucleoid structuring (H-NS) protein-encoding gene (BCAL0154) reduced bacterial accumulation and attenuated virulence in *C. elegans*. Further analysis using quantitative RT-PCR indicated that BCAL0154 modulates *B. cenocepacia* pathogenesis via transcriptional regulation of motility-associated genes including *fliC, fliG, flhD*, and *cheB1*. This screen has successfully identified genes required for *B. cenocepacia* survival within the host-associated environment, many of which are potential targets for developing new antimicrobials.

## Introduction

*Burkholderia cepacia* complex (Bcc) is a diverse group of Gram negative bacteria, all of which are ubiquitously distributed in a wide-range of ecological niches (Coenye and Vandamme, 2003). Bcc members are potentially beneficial for biological control of plant diseases and bioremediation. However, some species cause chronic respiratory infection in immunocompromised individuals particularly in patients with cystic fibrosis (CF) (Mahenthiralingam et al., 2005). *B. cenocepacia*, along with *B. multivorans*, are the predominant Bcc clinical isolates associated with infection in CF patients (Loutet and Valvano, 2010). Although the incidence rate of infection by Bcc is relatively low compared to other CF-associated pathogens (e.g., *Pseudomonas aeruginosa*), long-term pulmonary colonization by Bcc can lead to rapid deterioration of lung function which results in a fatal necrotizing pneumonia known as “*cepacia* syndrome” (Mahenthiralingam et al., 2005). Bcc members appear to be intrinsically resistant to many clinically available antimicrobials and treatment choices are limited (Loutet and Valvano, 2010).

To understand the Bcc mode of pathogenesis, virulence determinants that contribute to bacterial pathogenicity have been extensively studied (Loutet and Valvano, 2010). Nevertheless, molecular mechanisms that underlie the ability of this pathogen to survive and adapt in its infected host whilst establishing long-term persistence, are incompletely understood. To succeed in persistent colonization of its host, *B. cenocepacia* must be able to withstand various stresses exerted by the host environment, including host immune defenses, oxygen limitation and nutrient availability. Several studies have provided insights into the adaptive strategies exploited by *B. cenocepacia* to survive and proliferate during infection in different hosts. Hunt et al. (2004) employed signature-tagged mutagenesis (STM) to simultaneously screen a collection of transposon mutants in a rat model of chronic lung infection and uncovered 102 genes required for *B. cenocepacia* K56-2 survival. Global transcriptional profiling identified differentially expressed genes during infection of mouse macrophages and also in a rat chronic respiratory infection model (O’Grady and Sokol, 2011; Tolman and Valvano, 2012), including genes associated with bacterial motility, type VI secretion system as well as CepIR quorum sensing system. The nematode *Caenorhabditis elegans* is an alternative host model which has been routinely used to study Bcc pathogenicity and host-pathogen interactions (Huber et al., 2004; Ramos et al., 2012; Schwager et al., 2013). *B. cenocepacia* can establish a persistent intestinal infection in *C. elegans* (Huber et al., 2004) and the use of this *Bcc*-infection model has facilitated the discovery of several Bcc virulence factors, for example the nematocidal protein AidA (Huber et al., 2004) and the RNA chaperone Hfq (Ramos et al., 2012), as necessary for efficient bacterial colonization in the nematode gut.

Advances in DNA sequencing technology have led to the routine use of transposon sequencing approaches such as Transposon-Directed Insertion Sequencing (TraDIS) (Langridge et al., 2009) to simultaneously assay the role of every gene within a bacterial genome, under particular growth conditions. Recent Tn-seq and INSeq studies conducted under *in vivo* selective environments such as the lung of a murine model have facilitated the identification of *B. pseudomallei* (Gutierrez et al., 2015) and *Acinetobacter baumannii* (Wang et al., 2014) virulence factors, demonstrating that these tools are not limited to *in vitro* experimental settings. These high-throughput screens allow a comprehensive search for novel virulence determinants, providing insights into how bacteria evolve different host-adaptive strategies to establish a persistent infection that can ultimately manifest as acute or chronic disease.

Previously, we described the construction of a large-scale *B. cenocepacia* J2315 transposon mutant library consisting of approximately 500,000 mutants and identified the essential genome of *B. cenocepacia* required for *in vitro* growth (Wong et al., 2016). In this study, we sought to uncover genes required for *B. cenocepacia* survival in an *in vivo* environment. The analysis identified a total of 178 genes that encode proteins predicted to be essential for *B. cenocepacia* survival in a *C. elegans* infection model. Validation using single-gene deletion mutants reinforced the importance of selected genes in establishing an active infection in *C. elegans*. Our results provide an overview of what is required for *B. cenocepacia* to successfully persist in a nematode host, facilitating better understanding of the biology of *B. cenocepacia* in relation to adaptation to different ecological niches, as well as presenting a catalog of potential gene targets for antimicrobial drug development.

## Materials and Methods

### Bacterial and *C. elegans* Strains and Growth Conditions

*Burkholderia cenocepacia* strain J2315 was obtained from the BCCM/LMG Bacteria Collection, Belgium (strain number LMG 16656). *B. cenocepacia* transposon mutants used as the input pool were generated in our previous study (Wong et al., 2016). *B. cenocepacia* wild type and mutant strains were routinely grown in LB broth or Brain Heart Infusion (BHI) broth at 37°C with agitation. When appropriate, antibiotics used for selection were tetracycline (500 μg/mL) and gentamycin (4 μg/mL). *Escherichia coli* mobilizer strain RHO3 (López et al., 2009) was maintained in Luria-Bertani (LB) medium supplemented with 400 μg/mL diaminopimelic acid (Sigma-Aldrich, United States). The *C. elegans* strain *rrf3(pk1426);glp-4(bn2)* was obtained from the Tan Laboratory at Stanford University, United States. Worms were propagated and maintained at 16°C on nematode growth medium (NGM) agar plates seeded with a lawn of the standard laboratory food source, *E. coli* OP50. Age synchronization of the worms was conducted by bleaching with alkaline hypochlorite and sodium hydroxide to release embryos. For infection assays, worms were grown at 25°C until they reached young adult stage before they were used for infection.

### Infecting *C. elegans* With *B. cenocepacia* J2315 Transposon Mutants

Transposon mutants (∼1 × 10^10^ CFU) (input pool) were inoculated into 50 mL LB broth and incubated for 16 h at 37°C. An aliquot of 100 μL of the overnight culture was spread evenly on NGM agar in 6 cm Petri dishes and an additional 5 mL of the culture was reserved as output-LB. The NGM plates were incubated at 37°C for 24 h and allowed to equilibrate to room temperature for 24 h before use. The bacterial lawn was washed from one or two plates and collected as output-NGM. To establish an infection assay, age-matched young adult worms [*rrf3(pk1426);glp-4(bn2)*] (estimated to be at least 5,000 worms) were transferred directly onto the NGM agar pre-seeded with input pool mutants. Plates were incubated at 25°C throughout the infection assay (Supplementary Figure 1).

### Harvesting Mutants From *C. elegans*

Mutants were collected from worms at 6 and 24 hpi using the method described by Ooi et al. (2012) with minor modifications. At each time point, worms were washed with M9 buffer and transferred to microcentrifuge tubes. Each tube was centrifuged briefly at low-speed to remove the wash buffer. Worms were then anesthetized in 25 mM levamisole (Sigma-Aldrich, United States), washed three times in 200 μL antibiotic cocktail (25 mM levamisole and kanamycin 1,000 μg/mL), followed by incubation for 2 h to kill and minimize the bacterial cells associated with the worm cuticle. Then, worms were washed four times with 200 μL of 25 mM levamisole to remove the killed bacteria and residual antibiotic. Using a motorized pestle, worms were homogenized by mechanical disruption in 100 μL of 1% Triton X (Sigma-Aldrich, United States). To estimate the number of mutants recovered from each sample, serial dilutions were performed on the worm lysates. Ten microliters of each dilution was spotted on Ashdown agar. The remaining lysate was spread on modified LB agar (3% tryptone, 1% yeast extract, and 1.5% bacteriological agar) containing tetracycline and incubated for 24 hpi at 37°C. Colonies were washed with LB broth, scraped off and collected as *in vivo* output pools (output-6 hpi and output-24 hpi) (Supplementary Figure 1). Four separate experiments were conducted.

### Preparation of TraDIS Sequencing Libraries

Sequencing libraries of all four output pools (output-LB, output-NGM, output-6 hpi, and output-24 hpi) were prepared as previously described (Wong et al., 2016). Briefly, 1 μg of genomic DNA was sheared to fragments of 100–1,500 bp, followed by end repair and A-tailing with the NEBNext DNA library preparation kit (New England Biolabs, United States). Adapter was prepared by annealing together two oligos, MP_Ad_a and MP_Ad_b. The adapter was ligated to the A-tailed DNA fragments and 200 ng of the adapter-ligated DNA fragments was amplified over 22 cycles using the primers Tra_Fp and Tra_Mp_Rp which contain unique 7-bp indexes (indicated by N) for sample multiplexing (Meyer and Kircher, 2010). Amplified libraries were size selected (200–500 bp), purified and quantified on the Bioanalyzer and by quantitative real-time PCR using primers qPCR_P5 and qPCR_P7. Prepared libraries were sequenced on the Illumina HiSeq 2000 platform with the custom sequencing primer Tra_SeqP and index primer Tra_IndP. All primer sequences and PCR cycling conditions are provided in Supplementary Table 1.

### Bioinformatics Analysis

Sequence reads from the Illumina FASTQ files were filtered for 10 bases matching the 3′ end of the transposon (TAAGAGACAG) with one mismatch allowed. Non-tagged reads were discarded prior to sequence mapping. The transposon tag was removed from the matching reads and the edited reads were then mapped to the *B. cenocepacia* J2315 reference sequence (EMBL accession numbers AM747720, AM747721, AM747722, and AM747723) using SMALT-0.7.2. Reads that mapped to more than one position (non-unique) were excluded. The precise transposon insertion site was determined using Bio::Tradis^1^. Gene essentiality was assessed as previously described (Langridge et al., 2009; Wong et al., 2016). TIS located within the sequence spanning the 5–90% portion of each locus were analyzed. Log_2_ likelihood ratios (LLR) were calculated between the essential and non-essential models and a gene was classified as essential if it had a LLR of ≤2. Similarly, a gene was assigned as non-essential if it had a LLR of >2. To compare the differences in read abundance between the input pool and *in vivo* output pools, the log_2_ fold change ratios of the observed reads were calculated based on Langridge et al. (2009). For every gene (g), the log_2_ fold change is calculated as log_2_[(n_g,A_ + 100)/(n_g,B_ + 100)], where n_g,A_ is the number of observed reads in the output pool and n_g,B_ is the number of reads for the input pool (Wong et al., 2016). The correction of 100 reads smoothes out the high scores for genes with very low numbers of observed reads. Similarly, a normal mode was fitted to the mode of distribution of log_2_ fold change and *P*-value was calculated for each gene based on one-sided test. *P*-values were then corrected by the Benjamini-Hochberg adjustment method (Benjamini and Hochberg, 1995). Genes with log_2_ fold change of ≤2 and corrected *P*-value of <0.05 were considered to be essential for bacteria to grow under a particular condition.

The search against available essential genes within the Database of Essential Genes (DEG)^2^ (version 14.7, updated on October 24, 2016) (Luo et al., 2014) was performed using BLASTP with the default parameters provided in DEG. BLAST similarities at protein-protein level that resulted in E-values of 10^-10^ or less, were considered matches. *B. cenocepacia* virulence factors were obtained from the Virulence Factors Database (VFDB)^3^ (updated on February 11, 2017) (Chen et al., 2005) and the *Burkholderia* Genome Database^4^ (Winsor et al., 2008).

### Construction of Knockout Mutants

Deletion mutants of target genes BCAL0154, BCAL3116 and BCAL3135 were generated using the method described by López et al. (2009). PCR primers were designed to amplify the sequences upstream (US) and downstream (DS) of the targeted region. The forward primer for the amplification of the DS fragment included an oligonucleotide tail homologous to the 3′ end of the US fragment. All primer sequences are listed in Supplementary Table 1. After obtaining the US and DS fragments of each target gene, both fragments (US + DS) were joined by a subsequent round of PCR using the respective US forward and DS reverse primers, producing a new amplicon with a deletion between the US and DS fragments. All mutant constructs were then cloned into pGEM-T Easy Vector (Promega, United States) and transformed into *E. coli* DH5α. White colonies were selected on 5-bromo-4-chloro-3-indolyl-β-D-galactopyranoside (X-Gal) (Promega, United States) plates containing 100 μg/mL ampicillin. Colony PCR was performed using the US forward and DS reverse primers to confirm the insertion before further validation by DNA sequencing.

The confirmed mutant construct was released from the pGEM-T vector using appropriate restriction enzymes and cloned into the non-replicative plasmid, pEXKm5 (López et al., 2009). The pEXKm5-mutant constructs were transformed into *E. coli* RHO3 and white colonies were selected on X-Gal indicator medium containing 35 μg/mL kanamycin. Recombinant pEXKm5-mutant plasmids were delivered into *B. cenocepacia* J2315 via conjugal transfer (López et al., 2009) and positive transformants were selected on LB agar containing 1,500 μg/ml kanamycin and 50 μg/mL 5-bromo-4-chloro-3-indolyl-β-D-glucuronide (X-Gluc) (Gold Biotechnology, United States). Blue Km^R^ colonies are merodiploid clones resulting from the integration of the allelic replacement construct into the recipient bacterial chromosome by homologous recombination between cloned and chromosomal sequences. Merodiploids were confirmed using US forward and DS reverse primers.

To resolve merodiploids, clones were streaked onto yeast extract tryptone agar containing 15% sucrose and incubated at 25°C for at least 72 h. Single colonies were selected and streaked on both blank and kanamycin-supplemented LB agar. Kanamycin sensitive clones generated from a second homologous recombination event were selected. These Km^S^ clones are either wild type or mutant strains and could be distinguished using the US forward and DS reverse primers (flanking the mutant deletion regions). Mutants yielded smaller DNA fragments for the deleted region as compared to wild type. PCR using primers amplifying oriT of the pEXKm5 plasmid backbone was performed to demonstrate the absence of the pEXKm5 plasmid backbone in the mutant clones. Sanger sequencing was performed as a final verification step to confirm the successful deletion of the targeted region.

### Colony Forming Unit (CFU) Assay

To evaluate the ability of the *B. cenocepacia* mutants to colonize compared to the wild type strain, the CFU assay was performed. Ingested bacteria were harvested from infected worms using a method similar to the TraDIS screen described above but with minor modifications. Briefly, overnight cultures of the *B. cenocepacia* mutants and wild type were adjusted to an optical density (OD_595nm_) of ∼ 1.5 to standardize the initial inoculum. Twenty microliters of each bacterial suspension was spread evenly on NGM agar in 3.5 cm Petri dishes and incubated as previously described. Age-matched young adult worms were deposited onto NGM agar pre-seeded with individual strains. At 6, 24, 48, and 72 hpi, 10 worms were picked from each plate and transferred into microcentrifuge tubes containing 100 μL of 25 mM levamisole. A total of 3 tubes (technical replicates), each consisting 10 worms, were collected for each bacterial strain. After performing all the washing steps, the number of worms retained in each tube was recorded prior to homogenization in 50 μL of 1% Triton X using a motorized pestle. Worm lysates were serially diluted and spotted on Ashdown agar (in triplicate). Colonies were counted after 48 h of incubation at 37°C. The number of bacteria per worm was calculated by dividing the total number of bacterial CFU in 50 μL of worm lysate with the number of worms retained in each tube.

### Growth Curve Analysis

*Burkholderia cenocepacia* J2315 wild type and mutant strains were cultured in LB broth at 37°C for 16 h. Overnight cultures were adjusted to an OD_595nm_ of 0.5 in LB, M9 minimal medium and liquid NGM. A total of 500 μL was transferred into 50 mL fresh media (LB or M9 minimal) and 50 μL was transferred into 50 mL fresh liquid NGM. All bacterial cultures were incubated at 37°C with shaking at 250 rpm. At selected time points, aliquots of bacterial cultures were serially diluted to enumerate the number of live bacteria. For each dilution, 10 μL was dropped onto Ashdown agar (in triplicate) and plates were incubated for 48 h at 37°C.

### Motility Assay

Swimming and swarming were assessed using agar plate assays as previously described (Déziel et al., 2001) with minor modifications. Swim agar plates were composed of 1% tryptone, 0.5% NaCl, 0.3% agar whilst 0.5% agar plates were used to observe swarming. Bacterial cultures grown overnight in LB were adjusted to OD_595nm_= 0.5. One microliter of the OD-adjusted bacterial suspension was point inoculated onto the middle of three swimming or swarming plates and incubated at two temperatures, 25°C and 37°C. Plates were observed every 24 h and evidence of motility was taken as the presence of bacterial growth around the point of inoculation. The widest diameter of the circular turbid zones (swimming) and migratory growth pattern (swarming) were quantitatively measured.

### Total RNA Isolation and Quantitative RT-PCR (qRT-PCR)

Overnight cultures were diluted 1:100 in 20 mL LB broth and grown at 25°C until mid-logarithmic phase (OD_595nm_ of 0.4-0.6). Total RNA was isolated from two biological replicates of both wild type J2315 and the knockout mutant ΔBCAL0154 using TRIzol (Invitrogen) and residual DNA was removed with RNase-free DNase (Qiagen). qRT-PCR reactions (10 μL) were performed with purified RNA using the iTaq^TM^ One-Step RT-PCR kit with SYBR green detection according to the manufacturer’s instructions (Bio-Rad Laboratories) on the Bio-Rad CFX96 Touch Real-Time PCR Detection System, with primers at a final concentration of 300 nM and a data acquisition temperature of 76°C. BCAL0421 (*gyrB*) encoding DNA gyrase subunit B and BCAM0918 encoding sigma factor SigE were used as the reference standards (O’Grady et al., 2009; Peeters et al., 2010) as the expression of *gyrB* and *sigE* did not vary significantly (*p* > 0.05; unpaired, two-tailed Student’s *t*-test) between wild type and ΔBCAL0154 mutant (Supplementary Figure 2). All primer sequences are listed in Supplementary Table 1.

### Semi-Quantitative Biofilm Formation Assay and SEM Analysis

Biofilm quantification assay was conducted using the microtiter plate technique (Merritt et al., 2005). Overnight cultures were diluted to achieve a reading of OD_595_ = 1. Each OD-adjusted bacterial suspension (200 μL) was dispensed into 8 wells of a 96-well flat-bottom polystyrene microtiter plate (Greiner Bio-One) and incubated at 37°C for 48 h. Uninoculated BHI broth served as the negative control. Following incubation, the wells were carefully washed twice with 1X phosphate buffered saline (PBS) to remove non-adherent bacteria. The wells were then fixed with 99% (v/v) methanol for 15 min and air-dried, prior to staining with 200 μL filtered 2% crystal violet for 5 min. Excess stain was washed three times with water. The crystal violet bound bacterial cells were solubilized with 200 μL of 95% (v/v) ethanol and the released stain was measured at 570 nm using a microplate reader (Sunrise, Tecan, Switzerland). Two independent experiments were conducted.

### Nematode Killing Assay

Nematode killing assay was performed as described (Huber et al., 2004) with minor modifications. Briefly, overnight cultures of the *B. cenocepacia* strains were adjusted to OD_595nm_ = 1.5 and 20 μL of each bacterial suspension was spread evenly on NGM agar in 3.5 cm Petri dishes are incubated as previously described (see section “Infecting *C. elegans* With *B. cenocepacia* J2315 Transposon Mutants”). Forty age-matched young adult worms were laid onto the bacterial lawn (triplicate plates for each test strain). Survival of the worms was monitored by scoring the number of live and dead worms every 4–6 h interval. Nematodes were considered dead when they failed to respond to touch. Assays were performed at 25°C and *E. coli* OP50 was used as a control in the assays.

### Statistical Analysis

Colony forming unit (CFU) numbers were analyzed using GraphPad Prism version 5.04 (GraphPad Software) and statistical significance was determined using the Mann–Whitney *U*-test. For growth curves, doubling time (g) was calculated from the exponential phase using the formula: g = t log_2_/(log N_t_ – log N_0_) where N_0_ = number of CFU at a point during log phase, N_t_ = number of CFU at a different time point during log phase, and t = time interval between N_0_ and N_t_. The data from *in vitro* growth analysis, motility and biofilm formation assays were expressed as mean ± standard error of the mean (SEM) from at least two independent assays. Statistical analyses for data derived from growth analysis, motility and biofilm formation assays were performed using the unpaired, two-tailed Student’s *t*-test. Results obtained from worm killing assays were analyzed using the Kaplan–Meier non-parametric survival analysis in Statview^®^ 5.0.1 (SAS Institute, Inc.). The data were presented as mean ± standard deviation (SD) of a representative from at least three independent experiments.

### Nucleotide Sequence Accession Numbers

Sequence reads were deposited in the European Nucleotide Archive database with accession number PRJEB13677 and are accessible via http://www.ebi.ac.uk/ena/data/view/PRJEB13677. The sample accession numbers are ERS1124791 (LB), ERS1124792 (NGM), ERS1124793 (6 hpi), and ERS1124794 (24 hpi).

## Results

### *In Vivo* Screening of a Transposon Mutant Pool in *C. elegans*

The large-scale *B. cenocepacia* transposon mutant pool (input pool) previously generated (Wong et al., 2016) was used as the initial inoculum for *C. elegans* infection. The input pool was first grown in Luria-Bertani (LB) medium before spreading on NGM, a minimal medium commonly used for nematode slow killing assays. At this juncture, we considered the likelihood that these bacterial preparation steps could introduce selection pressure on the mutant population resulting in the loss of some mutants from the initial pool prior to subjecting the input pool to *in vivo* selection. Hence, to assess potential changes to the *in vitro* grown population, we harvested mutants grown in LB and NGM that are hereafter referred to as output-LB and output-NGM.

A limitation of this infection assay is probably the restricted number of mutants ingested and subsequently allowed to enter the worm. To determine the logarithmic phase in which the ingested bacteria are actively proliferating, a CFU assay was performed to enumerate the number of *B. cenocepacia* at 6-, 24-, 48-, and 72-hpi. The number of wild type bacteria accumulated in the worm increased from 6 to 24 hpi and remained constant up to 72 hpi, i.e., ∼10^4^ CFU per worm (Supplementary Figure 3). As the number of accumulated *B. cenocepacia* in the infected worms was maximum in the first 24 h, bacteria were recouped at 6 and 24 hpi to represent: the initial logarithmic phase of bacterial growth during early infection, and the maximal number of bacteria residing in the worm, respectively. As the number of bacteria that can reside in a single worm is most likely lower than the total number of individual mutants (∼5 × 10^5^ mutants) present in our input pool, we performed a large-scale infection assay using at least 5,000 worms per experiment. At each time point (6 and 24 hpi), bacterial mutants were recovered from a population of worms. We conducted four separate experiments and all mutants obtained were pooled, resulting in a total of approximately 1 × 10^7^ mutants for each *in vivo* output pool (Supplementary Figure 1).

Next, TraDIS libraries of four output pools (LB, NGM, 6 and 24 hpi) were prepared as previously described (Wong et al., 2016). For each TraDIS library, we generated an average of 25 million reads, with >96% of the total reads containing the appropriate 10 bp transposon tag. Over 68% of the tagged reads were mapped to the reference genome, resulting in between 128,000 and 500,000 unique Transposon Insertion Sites (TIS) identified across the genome (Supplementary Table 2). For output-6 hpi and output-24 hpi libraries, one TIS was detectable every 44 bp and for each insertion, more than 50 reads were routinely obtained. We believe this high density of transposition events provides sufficient coverage to estimate the relative levels of mutant abundance before (input pool) (Wong et al., 2016) and after selection (output pool). For each pool, read counts and fold change values for all genes are listed in Supplementary Data Table 1D.

### Genes Required for *B. cenocepacia* Survival *in Vivo* During the Initial 24 h of Infection

First, we identified the conditionally essential genes required to sustain bacterial *in vitro* growth in LB and NGM (Supplementary Data Table 1A). Insertions in genes essential for *in vitro* growth are likely to reduce the population of resulting loss-of-function mutants initially present in the input pool used for infection. Hence, in the context of this study, *in vitro* selection pressure was taken into consideration when analyzing the output pools.

**FIGURE 1:**
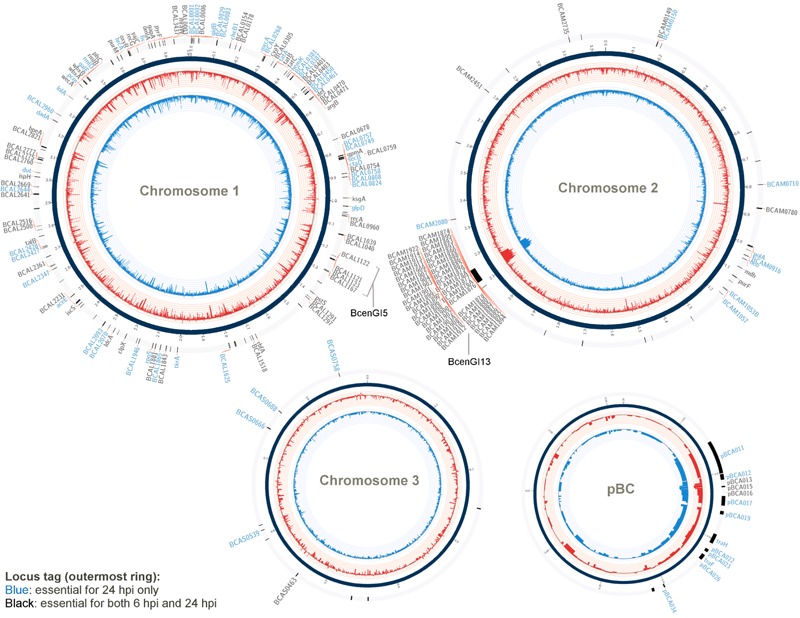
Genes required for *B. cenocepacia* for *in vivo* survival. A Circos-derived (Krzywinski et al., 2009; Cheong et al., 2015) atlas representation of *Burkholderia cenocepacia* J2315 genome is shown (each chromosome is not drawn in scale). Outer ring (track 1; arranged clockwise) shows the chromosomal locations of the genes predicted as essential for *in vivo* survival at selected time points (locus tag labeled blue: essential for 24 hpi only; black: essential for both 6 and 24 hpi). The log_2_ fold-change level (relative reads abundance in the *in vivo* output pools compared to that in the input pool) is depicted by the histogram in the inner-most two rings of the circular maps (track 2 and 3). Red bars (track 2) represent data obtained for output-6 hpi; blue bars (track 3) represent data obtained for output-24 hpi. The height of the histogram bars corresponds to the degree of fold-change level (log_2_-transformed).

A gene was considered to be essential for *B. cenocepacia* survival and proliferation in the *C. elegans* (i.e., *in vivo* fitness) if the relative reads abundance was at least four times significantly lower in the *in vivo* output pool (log_2_ fold change ≤2) than that in the initial input pool (adjusted *p*-value < 0.05). A total of 271 genes from output-6 hpi and 296 genes from output-24 hpi met this analysis criterion (Supplementary Data Table 1B). When determining the set of genes required for *B. cenocepacia* survival *in vivo*, we excluded conditionally essential genes necessary for *in vitro* growth (LB and NGM). As a result, a total of 171 genes were deemed important for *B. cenocepacia* survival at 6 hpi and 178 genes were identified for 24 hpi. In comparing both gene sets, we noted that 113 genes are required for *B. cenocepacia in vivo* survival at both time points (6 and 24 hpi). A total of 58 genes were only detected at 6 hpi but not 24 hpi (Supplementary Data Table 1C). This could be explained either by the fact that the capacity of these insertion mutants to proliferate recovers after prolonged infection or that these mutants were not consumed by the worms during the initial 6 hpi. Thus, we excluded the subset of genes that were only detected at 6 hpi. Altogether, our TraDIS analysis predicted a total of 178 genes (about 3% of the *B. cenocepacia* J2315 genome) that are required for *B. cenocepacia* survival in the *C. elegans rrf3(pk1426);glp-4(bn2)* strain (**Figure 1** and Supplementary Data Table 2).

**FIGURE 2:**
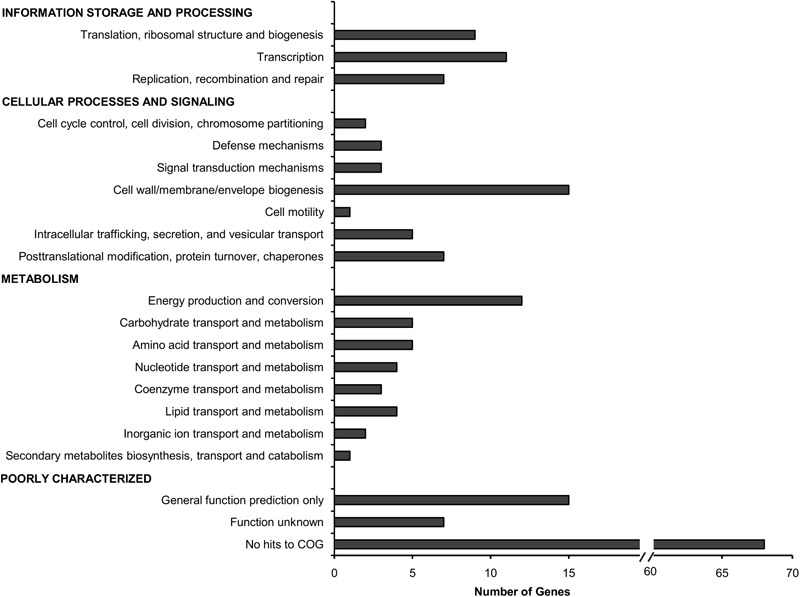
Functional distribution of genes required for *B. cenocepacia in vivo* survival in *C. elegans*. The number of genes for which Tn*5* insertions resulted in decreased read abundance in the *in vivo* output pools relative to the input pool (log_2_ fold change ≤2; adjusted *p*-value < 0.05) in each COG category is shown.

The total 178 TraDIS-derived candidates (Supplementary Data Table 2) were classified based on the Cluster of Orthologous Groups (COG) annotation system (**Figure 2**). Approximately 25% of the genes deemed essential for *B. cenocepacia in vivo* survival were functionally classified into the categories of “cell wall/membrane/envelope biogenesis” (15/178), “transcription” (11/178), “energy conversion and metabolism” (12/178) and “replication, recombination and repair” (7/178). Six TraDIS-derived genes were also identified by STM as essential for *B. cenocepacia in vivo* survival in a rat model of chronic lung infection (Hunt et al., 2004) (Supplementary Data Table 2). Lack of similar functional genes may suggest that *B. cenocepacia* can adopt distinct adaptive strategies to survive in different host environments, in this case, the intestinal lumen of *C. elegans* and the lung of an infected rat. A similar observation was noted for mycobacteria whereby *M. marinum* uses host-specific mechanisms to adapt to different intracellular environments (Weerdenburg et al., 2015).

Of 178 genes required for *B. cenocepacia in vivo* survival, we identified six genes located in the LPS *O*-antigen biosynthesis gene cluster (**Table 1**). This is consistent with that reported by Hunt et al. (2004), where an STM-based screen isolated genes involved in LPS *O*-antigen synthesis necessary for *B. cenocepacia* survival *in vivo* in a rat model. The presence of *O*-antigen and its role in virulence, including serum resistance, modulation of macrophage phagocytosis as well as bacterial adherence, has been described in other *Burkholderia* species (DeShazer et al., 1998; Saldías et al., 2009). Given that *B. cenocepacia* strain J2315 has lost its ability to produce LPS with a complete *O*-antigen (Ortega et al., 2005), these *O*-antigen biosynthesis genes may be involved in other biological functions that are important for bacterial survival as well as virulence.

**Table 1:** Selected *B. cenocepacia* genes identified by TraDIS *in vivo* screen.

Gene ID	Gene	Function	Log_2_ fold change	
			6 hpi	24 hpi
*LPS O-antigen biosynthesis*				
BCAL3115	*wbxA*	Glycosyltransferase	-5.08	-3.67
**BCAL3116**	***wbxB***	**Glycosyltransferase**	**-4.54**	**-2.89**
BCAL3117	*galE*	UDP-glucose epimerase		-3.01
BCAL3118	*wecA*	UDP-*N*-acetylglucosamine-1-P transferase	-4.39	-3.24
BCAL3132	*rmlD*	dTDP-4-keto-L-rhamnose reductase		-3.41
**BCAL3135**	***rmlB***	**dTDP-D-glucose 4,6-dehydratase**	**-4.77**	**-4.09**
*Stress response*				
**BCAL0154**		**Histone-like nucleoid-structuring (H-NS) protein**	**-5.65**	**-5.06**
BCAL0953	*recA*	Recombinase A	-4.07	-4.45
BCAL1892	*rpoS*	RNA polymerase sigma factor RpoS		-2.91
BCAL1995	*clpX*	ATP-dependent protease ATP-binding subunit ClpX	-4.02	-4.97
BCAL3275	*hrcA*	Heat-inducible transcription repressor		-3.27
BCAL3301	*oxyR*	Oxidative stress regulatory protein	-6.18	-4.89
BCAL3302	*recG*	ATP-dependent DNA helicase RecG	-7.22	-5.19
BCAL3335	*fis*	DNA-binding protein Fis		-3.16
*Secretion system*				
BCAL0323	*tatA*	Twin arginine translocase protein A		-4.01
BCAL0324	*tatB*	Sec-independent translocase	-4.70	-3.38
BCAL0325	*tatC*	Sec-independent protein translocase protein TatC	-5.65	-3.42
BCAL0742	*secB*	preprotein translocase subunit SecB		-2.90
BCAL3305	*yajC*	preprotein translocase subunit YajC	-5.53	-3.05
*Energy metabolism*				
BCAL0749		Putative cytochrome c oxidase		-3.25
BCAL0750	*ctaD*	Cytochrome c oxidase polypeptide I		-3.64
BCAL0754		Putative cytochrome c oxidase subunit III	-4.03	-3.67
BCAL0758		Putative cytochrome oxidase assembly protein		-4.07
BCAL0759		Protoheme IX farnesyltransferase	-3.44	-4.54
*Iron uptake and metabolism*				
BCAL0273	*cyaY*	Frataxin-like protein	-4.59	-3.81
*Chemotaxis and motility*				
BCAL0134	*cheB1*	chemotaxis-specific methylesterase		-3.18
*Antimicrobial resistance*				
BCAL2821		RND family efflux system transporter protein	-5.92	-6.11
BCAL2822	*bpeA*	Efflux system transporter protein	-5.37	-5.08
BCAL0274	*mrcA*	Penicillin-binding protein 1A		-2.95

*Genes for which mutant abundance was significantly reduced in the *in vivo* output pools (6 and 24 hpi) compared to the initial input pool are predicted to be required for *in vivo* survival in *C. elegans* (log_*2*_ fold change less than -2, *p*-value < 0.05). Genes highlighted in bold were selected for deletion mutant construction.*

Our screen also identified a number of genes encoding proteins required for general stress response, e.g., RpoS, a key response regulator to starvation, heat and oxidative stress in *B. cepacia* and *B. pseudomallei* (Aguilar et al., 2003; Subsin et al., 2003). Histone-like nucleoid-structuring (H-NS) protein is often referred to as a “universal repressor” and in many bacteria, H-NS family members appear to be involved in regulating bacterial pathogenicity (Dillon and Dorman, 2010). The OxyR transcriptional regulator modulates expression of many genes associated with oxidative stress defense (Peeters et al., 2010), including genes encoding peroxide scavenging enzymes (e.g., catalase KatB). *C. elegans* can respond to pathogens by generating reactive oxygen species (ROS) in the intestine while simultaneously instigating an oxidative stress response to protect adjacent tissues (Chavez et al., 2007), hence, under *in vivo* conditions, *B. cenocepacia* is likely to require OxyR for efficient detoxification of antibacterial ROS. Given that the nematode intestine is a metabolically active organ, it is conceivable that ingested *B. cenocepacia* require fine-tuning of their aerobic respiration. Genes encoding the *aa_*3*_* type heme-copper cytochrome *c* oxidase subunits (BCAL0749, BCAL0750, and BCAL0754) were also identified. The *aa_*3*_* type heme-copper cytochrome *c* oxidase is one of the terminal oxidases that reduce oxygen to water during the aerobic respiratory chain.

Disruption of genes encoding the major components of the twin arginine translocation (Tat) pathway (TatA, TatB, and TatC), which transports folded proteins across the bacterial membrane, reduced *B. cenocepacia* survival in *C. elegans*. TraDIS also identified genes that make up the *ptw* cluster (pBCA020-pBCA059), a plasmid-encoded type IV secretion system (T4SS) (Holden et al., 2009). This plasmid-borne T4SS secretes plant cytotoxic proteins that cause the plant tissue water-soaking (*ptw*) phenotype in onions (Engledow et al., 2004) and contribute to the increased survival of *B. cenocepacia* in macrophages and airway epithelial cells (Sajjan et al., 2008).

Approximately 51% of the identified genes (90/178) belong to the “poorly characterized” group, of which almost 80% have no COG classification (**Figure 2**). A majority of these genes with unknown function were localized within *B. cenocepacia* genomic islands (e.g., BcenGI5 and BcenGI13) as well as the pBC plasmid, suggesting that the factors contributing to bacterial *in vivo* survival may have been acquired via mobile genetic elements. The presence of these plasmid and GI-encoded genes shows strain-specific variation, for instance, the prophage BcenGI13 is absent in *B. cenocepacia* strain H111 which is not a member of the epidemic ET12 lineage (Supplementary Figure 4). Upon *B. cenocepacia* internalization into murine macrophages, transcripts of several genes located in BcenGI13 were detected by SCOTS (selective capture of transcribed sequences), indicating that these genes are preferentially expressed (Tolman and Valvano, 2012). Nonetheless, the functional role of BcenGI13 is not known, though it has been suggested that the presence of prophages among *Burkholderia* members may contribute to genomic variability and provide advantages in relation to niche adaptation (Ronning et al., 2010).

### Validation of TraDIS Results Using Single-Gene Knockout Mutants

The TraDIS screen identified a total of 113 genes as crucial for *B. cenocepacia in vivo* survival in *C. elegans* at both 6 and 24 hpi. To validate this screen, single-gene knockout mutants for three randomly selected genes, *wbxB* (BCAL3116), *rmlB* (BCAL3135) and BCAL0154, were constructed. Six genes located in the LPS *O*-antigen biosynthesis gene cluster were detected in our TraDIS screen and LPS *O*-antigen biosynthesis genes were also reported by Hunt et al. (2004). For the validation, we selected the *O*-antigen related genes *wbxB* (BCAL3116) and *rmlB* (BCAL3135). BCAL0154 encodes a H-NS-like transcriptional regulator and H-NS family members have been identified as regulators of virulence in bacteria such as *V. cholerae* (Nye et al., 2000) and uropathogenic *E. coli* (Müller et al., 2006), but not yet in *B. cenocepacia*. The fold change values of the three selected genes in TraDIS screen (mutant abundance in output-6hpi and output-24hpi relative to the input pool) were shown in **Table 1**.

**FIGURE 3:**
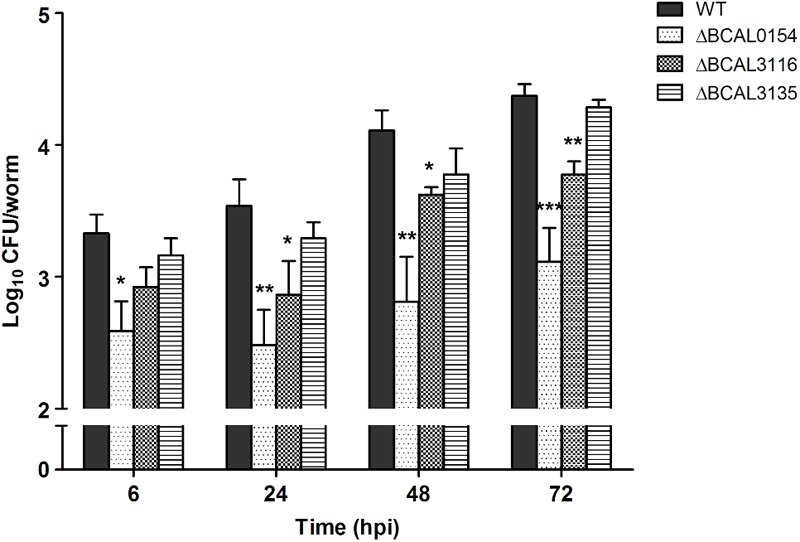
Bacterial counts for *B. cenocepacia* J2315 wild type and knockout mutants in *C. elegans*. The CFU number of *B. cenocepacia* knockout mutants ΔBCAL0154, ΔBCAL3116, and ΔBCAL3135 and their isogenic wild type (WT) strain recovered from the infected worms at different time points (6, 24, 48, and 72 hpi) are illustrated. The bars correspond to mean ± SEM of the number of CFU (log_10_) per worm from two independent experiments of three replicates each; in each replicate, ingested bacteria were extracted from 10 infected worms. The asterisks represent *P*-values of the WT counts versus the mutants (unpaired, two-tailed Student’s *t*-test; ^∗^*p* < 0.05; ^∗∗^*p* < 0.01; ^∗∗∗^*p* < 0.001). Throughout the infection period, the number of ΔBCAL0154 mutants recovered is significantly lower than that of the wild type strain. Disruption of BCAL3116 affected bacterial survival after 24 h of infection. At all-time points tested, ΔBCAL3135 accumulated in *C. elegans* to a level similar to wild type J2315.

The knockout mutants (ΔBCAL0154, ΔBCAL3116, and ΔBCAL3135) were assessed for their ability to infect *C. elegans* when compared to the wild type strain (WT). As shown in **Figure 3**, at 6 hpi, the number of viable ΔBCAL0154 mutants recovered from infected worms is significantly reduced (*p* < 0.01). At 24, 48, and 72 hpi, the bacterial load in worms infected with ΔBCAL0154 and ΔBCAL3116 was significantly lower when compared to worms infected with the wild type strain. This result concurs with our TraDIS-based prediction that the absence of BCAL0154 and BCAL3116 would impact *B. cenocepacia* survival in *C. elegans*. Deletion of BCAL0154 and BCAL3116 did not reduce bacterial *in vitro* growth in LB, M9 minimal medium and liquid NGM (Supplementary Figure 5). Hence, we ruled out the likelihood that reduced accumulation by ΔBCAL0154 and ΔBCAL3116 was due to general bacterial growth defects or increased sensitivity to nutrient limitation prior to worm infection.

The knockout mutant ΔBCAL3135, however, did not show any pronounced defect in inhabiting the worm. This contradicts our TraDIS-based observation where insertions in this gene caused up to 16-fold reduction in mutant abundance in *in vivo* output pools (Supplementary Data Table 2). In our TraDIS screen, worms were infected with a mixed population of transposon mutants; hence, it is conceivable that the outcome of the TraDIS-based prediction may not be similarly reflected in a single-strain infection experiment with a chosen mutant. We hypothesize that deletion of BCAL3135 does not directly lead to significant arrest of bacterial growth *in vivo*, but rather affects the overall competitive fitness of the resulting mutant in a mixed bacterial population.

### Phenotypic Characterization of Attenuated *B. cenocepacia* Knockout Mutants

**FIGURE 4:**
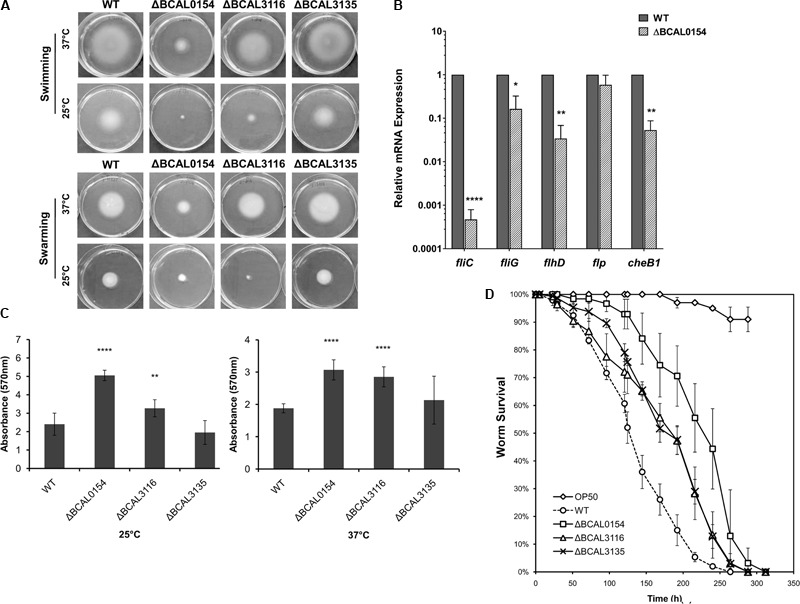
Phenotypic characterization of *B. cenocepacia* J2315 wild type (WT) and the knockout mutants ΔBCAL0154, ΔBCAL3116, and ΔBCAL3135. **(A)** Swimming and swarming at 25°C and 37°C. The images shown are representative of two independent experiments (for each experiment, three technical replicates were performed). The motility of ΔBCAL0154 and ΔBCAL3116 decreased, whereas no defect in motility was observed for ΔBCAL3135 (unpaired, two-tailed Student’s *t*-test). **(B)** Expression levels of motility-associated genes in ΔBCAL0154 relative to wild type. Data are mean ± SEM from two independent experiments with replicate measurements. The asterisks represent *P*-values of the WT expression levels versus ΔBCAL0154 (unpaired, two-tailed Student’s *t*-test; ^∗^*p* < 0.05; ^∗∗^*p* < 0.01; ^∗∗∗∗^*p* < 0.0001). **(C)** Biofilm formation at 25°C and 37°C. Each bar represents the mean OD_570nm_ value ± SEM obtained from two independent experiments; in each experiment, measurements of eight technical replicates were taken. At both temperatures tested, levels of biofilm produced by ΔBCAL0154 and ΔBCAL3116 were significantly higher (unpaired, two-tailed Student’s *t*-test; ^∗∗^*p* < 0.01, ^∗∗∗∗^*p* < 0.0001). **(D)** Survival of *C. elegans* infected with the deletion mutants and wild type J2315. Uninfected worms fed with *E. coli* OP50 served as a control. Graph shows the mean ± SD from a representative of three independent assays. In a pair-wise analysis using the log-rank test, ΔBCAL0154, ΔBCAL3116, and ΔBCAL3135 were significantly attenuated compared to wild type (Kaplan-Meier; *p* < 0.0001).

We evaluated the capability of knockout mutants in terms of motility, biofilm formation and virulence in *C. elegans.* As bacterial motility is an important trait required for successful host colonization (Ribet and Cossart, 2015), we investigated whether disruption of BCAL0154, BCAL3116, and BCAL3135 would abolish *B. cenocepacia* swimming and swarming in soft agar. At 37°C, the ΔBCAL0154 mutant was severely impaired in both swimming and swarming based on the reduction in swimming/swarming zones compared to the wild type strain (**Figure 4A**). The ΔBCAL3116 mutant showed a modest decrease in swimming but not swarming. In *E. coli*, H-NS regulates more than two-thirds of the temperature-regulated genes (White-Ziegler and Davis, 2009). We therefore asked if the motility defect exhibited by the ΔBCAL0154 mutant is visible at the nematode infection experimental temperature of 25°C. At this lower temperature, ΔBCAL0154 and ΔBCAL3116 displayed an obvious defect in both swimming and swarming (**Figure 4A**) which supports our earlier observation that these two mutants do not proliferate well in *C. elegans*. This suggests that motility is important for *B. cenocepacia* survival in a host and motility involves both the BCAL0154 and BCAL3116 gene products.

As H-NS family members often function as global transcriptional regulators (Dillon and Dorman, 2010), we hypothesized that the H-NS protein encoded by BCAL0154 modulates the expression of motility-associated genes. As depicted in **Figure 4B**, expression of flagella-related genes was significantly down regulated in ΔBCAL0154 relative to wild type, which correlates with the motility defects represented in **Figure 4A**. In particular, the expression of *fliC*, which encodes the major flagellin subunit, is reduced by more than 6,000-fold. Genes encoding the master regulator of flagella assembly FlhD and flagella motor switch protein FliG were also down regulated in ΔBCAL0154. In addition, loss of BCAL0154 reduced the expression of the chemotaxis gene BCAL0134 (CheB1), which we also predicted to be required for *B. cenocepacia in vivo* survival (**Table 1**).

*B. cenocepacia* is known to produce biofilm on cell surfaces (Coenye, 2010) in response to various forms of stress such as the presence of antibiotics. A reduction in biofilm formation could render the bacteria more sensitive to the host environment resulting in a lower number of proliferating bacteria. Surprisingly, both ΔBCAL0154 and ΔBCAL3116 produced significantly higher amounts of biofilm at both 37°C as well as the infection experimental temperature of 25°C (**Figure 4C**) (*p* < 0.01). We speculate that the increased biofilm formation by ΔBCAL0154 and ΔBCAL3116 may be associated with their altered swimming motility, although the relationship between biofilm formation and motility has not been described in *B. cenocepacia*.

*B. cenocepacia* is able to kill *C. elegans* via two killing modes, i.e., slow-killing and fast-killing, depending on the growth medium used (Köthe et al., 2003). On high-osmolarity medium, worm killing by *B. cenocepacia* involves the production of extracellular toxin which paralyzes the worms (fast killing). On NGM, slow killing and worm mortality is associated with intestinal bacterial accumulation (Köthe et al., 2003; Huber et al., 2004). As such, we examined whether BCAL0154, BCAL3116, and BCAL3135 are required for efficient killing of *C. elegans*. Worms were infected with the mutant strains and nematode survival was evaluated using the slow-killing assay. Individual deletion of BCAL0154, BCAL3116, and BCAL3135 significantly abrogated virulence in *C. elegans* (**Figure 4D**) as reflected by the mean times-to-death (Supplementary Table 3). Of the mutants tested, ΔBCAL0154 (H-NS-like transcriptional regulator) was strongly attenuated, implicating the possible role of BCAL0154 in regulating *B. cenocepacia* pathogenicity. The reduced virulence of ΔBCAL0154 and ΔBCAL3116 might be caused, at least in part, by their deficient motility and also decreased ability to survive in *C. elegans*. Although the ΔBCAL3135 mutant was attenuated, this was not reflected by a reduced accumulation of bacteria in the nematode. However, the function of the protein encoded by this gene in relation to *B. cenocepacia* pathogenesis remains unknown.

## Discussion

This study identified 178 genes that confer benefits to *B. cenocepacia* fitness in terms of survival in the model infection host *C. elegans*. These include genes involved in *de novo* biosynthesis of amino acids and nucleic acids, biosynthesis of LPS *O*-antigen, responses toward various forms of stress (e.g., oxidative stress) as well as defense against host immune effectors (e.g., RND efflux pumps).

In an earlier study, we identified 439 conditionally essential genes required for *B. cenocepacia* survival in a nutrient-limiting environment (M9 minimal medium) (Wong et al., 2016). Of these, 44 genes appear to be also required for *in vivo* survival (Supplementary Data Table 2) including genes encoding for Sec-independent protein secretion pathway components and key purine biosynthesis proteins phosphoribosylaminoimidazole synthetase (purM) and amidophosphoribosyltransferase (purF). Purine biosynthesis has been implicated in the virulence of *B. cenocepacia* H111 in *C. elegans* (Schwager et al., 2013). Identification of the purine biosynthesis genes *purF* and *purM*, imply that the internal environment of the worm is likely to be purine-restricted during *B. cenocepacia* infection. Genes involved in the biosynthesis of amino acids (i.e., *argB, talB, gpmA*, BCAL0401 and BCAL2231) were also identified suggesting that *B. cenocepacia* responds to nutritional cues within the host environment by adjusting its metabolic pathways to sustain growth.

Several studies have shown that the presence of LPS *O*-antigen enhances bacterial colonization in the nematode intestine as well as the eventual death of *C. elegans* (Browning et al., 2013; Kuo et al., 2016). Nevertheless, it is worth noting that *B. cenocepacia* strain J2315, while retaining the ability to colonize the *C. elegans* intestine (Ramos et al., 2012), produces an LPS that lacks a complete *O*-antigen due to the presence of an insertion element in the *wbxE* gene (Ortega et al., 2005). In light of this, we further investigated the possible roles of *O*-antigen biosynthesis genes *wbxB* and *rmlB* identified by our TraDIS screen in terms of *B. cenocepacia in vivo* survival and other virulence-associated phenotypes. Our study showed that deletion of *wbxB* (BCAL3116) which encodes a glycosyltransferase influenced *B. cenocepacia* accumulation, motility, biofilm formation and virulence in *C. elegans*. A *wbxCD* mutant lacking a putative acetyltransferase and an *O*-antigen glycosyltransferase is less motile, most likely a result of loss of flagellin glycosylation (Hanuszkiewicz et al., 2014). Hence, the non-motile phenotype of the *wbxB* mutant may also suggest a potential role for the WbxB protein in the regulation of flagella glycosylation.

On the other hand, *rmlB* (BCAL3135) which has been reported to be essential in *B. cenocepacia* strain K56-2 (Hanuszkiewicz et al., 2014), is not essential for strain J2315 viability (Supplementary Figure 6). RmlB (dTDP-D-glucose 4,6-dehydratase) is needed for the synthesis of nucleotide sugars like dTDP-L-rhamnose which constitute part of the *O*-antigen polysaccharide present in strain K56-2 but not the *O*-antigen-defective strain J2315 (Ortega et al., 2005). As *O*-antigen production is thought to be not required for *B. cenocepacia* viability, Hanuszkiewicz et al. (2014) raised the possibility that RmlB is involved in the biosynthesis of other nucleotide sugars that may play an essential role in a yet unknown metabolic pathway in strain K56-2. The essentiality of *rmlB* in strain K56-2 is further supported by a recent Tn-seq study (Gislason et al., 2017), which also identified distinct sets of genes that are uniquely essential in K56-2 and J2315. Nonetheless, while disruption of J2315 *rmlB* in our study did not affect mutant accumulation in the worm in a single-strain infection experiment, abolishment of RmlB led to reduced killing of *C. elegans*. Further work is needed to reveal the roles of these LPS-related genes in *B. cenocepacia* pathogenicity.

Interestingly, our study identified the BCAL0154 gene encoded histone-like nucleoid structuring (H-NS) protein to be potentially involved in the pathogenicity of *B. cenocepacia* in the *C. elegans* infection model. In a slow-killing assay, BCAL0154 deletion mutant exhibited attenuated *C. elegans* killing, which is most likely a reflection of the mutant’s inability to accumulate in the worm relative to infection by the wild type strain (**Figure 3**). An STM screen also identified a H-NS mutant as attenuated for *in vivo* survival in a rat infection model (Hunt et al., 2004). It is well documented that most nucleoid-associated proteins (NAPs) influence nucleoid structure and may function as global regulators of gene expression(Dillon and Dorman, 2010). Antagonistic interaction between H-NS and ToxR, a central regulator that affects gene expression within all *Vibrio* pathogenicity islands, controls *V. cholerae* host colonization and impacts biofilm formation (Kazi et al., 2016). In *E. coli*, H-NS acts to stimulate flagella gene expression and cells deleted for the *hns* gene are unable to assemble flagella (Paul et al., 2011). Likewise, our data showed that abolishment of BCAL0154 in *B. cenocepacia* resulted in diminished swimming and swarming ability, most likely due to the down-regulation of motility-associated genes such as *fliC*. The importance of flagella in bacterial pathogenicity is further supported by the reduced virulence of a *B. cenocepacia fliC* mutant in a murine model of infection (Urban et al., 2004). We propose that BCAL0154 contributes to *B. cenocepacia* virulence via transcriptional regulation of flagella genes. As inhibition of motility promotes biofilm formation (Guttenplan and Kearns, 2013), we showed that disruption of BCAL0154 also led to an increased level of biofilm, suggesting a possible role for H-NS in mediating planktonic to biofilm transition. In many other bacteria, mutations in H-NS encoding genes lead to pleiotropic phenotypes, ranging from diminished growth rates to induction of stress responses to altered expression of virulence factors (Müller et al., 2006; Wang et al., 2015). Collectively, these findings warrant the need for further work to clarify the regulation of BCAL0154 in the context of *B. cenocepacia* pathogenicity and adaptation in the infected host.

Juhas et al. (2012) reported the core genome of the order *Burkholderiales* which is made up of 610 orthologous groups that are present in the genomes of 51 *Burkholderia* species. Only 26% of our TraDIS-derived genes (47/178) are part of the *Burkholderiales* core genome, suggesting that genes required for accessory functions (i.e., *in vivo* survival) are not evolutionary conserved and could be acquired or lost during *B. cenocepacia* adaptation to different niches (Supplementary Data Table 2). We also identified 79 genes (44%) with homologs among genes deposited in the Database of Essential Genes (DEG) (Luo et al., 2014) (Supplementary Data Table 2). Genes that are absent in the core genome of *Burkholderiales* and have no homolog in DEG may play contributory roles in *B. cenocepacia* survival and persistence in distinct host niches.

Several gene products have been shown to influence colonization of *B. cenocepacia* in *C. elegans*, e.g., nematocidal protein AidA (Huber et al., 2004) and the RNA chaperone Hfq (Ramos et al., 2012). The *B. cenocepacia* AidA mutant was unable to colonize the intestinal lumen of *C. elegans* even when the worm pharyngeal grinder was defective, suggesting that this gene is required for survival and/or proliferation in the nematode gut rather than encoding a toxin (Huber et al., 2004). Conversely, our TraDIS screen did not identify this *aidA* gene as essential for *B. cenocepacia* survival *in vivo* in the worm. In *B. cenocepacia*, AidA synthesis is strictly mediated by AHL signal molecules, positively by CepIR quorum sensing and negatively by CciIR and CepR2 (Subramoni and Sokol, 2012). As the TraDIS screen involves a large pool of mutants competing to survive, it is plausible that any effect exerted on the individual AidA mutant bacteria may be compensated by other bacterial cells in the population.

It is not known to what extent the worm internal environment resembles the organs of higher eukaryotes, particularly a CF lung, but there is some evidence to suggest conservation in Bcc virulence mechanisms between the active infection of CF mice and the fast and slow killing of nematodes (Leitão et al., 2010; Uehlinger et al., 2009).

## Conclusion

We have demonstrated the application of TraDIS for *B. cenocepacia in vivo* selection and this approach can be exploited as a useful tool to investigate the molecular mechanisms that underlie *B. cenocepacia* versatility. More importantly, the identification of *B. cenocepacia* genes essential for fitness in *C. elegans* has provided an overview of *B. cenocepacia* adaptation to an *in vivo* setting. Whilst the identity of many of the genes deemed as essential for *B. cenocepacia* fitness is unknown, some may potentially encode for *B. cenocepacia* virulence factors that will serve as appealing targets for the development of new antimicrobials.

## Author Contributions

SN, AP, MA, and Y-CW conceived the project, designed the experiments, analyzed the data, and wrote the paper. Y-CW performed the experiments. RG, S-JY, and C-CH performed part of the bioinformatics analysis. All authors read and approved the final manuscript.

## Conflict of Interest Statement

The authors declare that the research was conducted in the absence of any commercial or financial relationships that could be construed as a potential conflict of interest.

## Footnotes

1 https://github.com/sanger-pathogens/Bio-Tradis

2 http://tubic.tju.edu.cn/deg/

3 http://www.mgc.ac.cn/VFs/

4 http://www.Burkholderia.com
