# Supplementary material for: Genetic Determinants Associated With in Vivo Survival of Burkholderia cenocepacia in the Caenorhabditis elegans Model
Source: Front Microbiol. 2018 May 29;9:1118. doi: 10.3389/fmicb.2018.01118 (PMC5987112; doi:10.3389/fmicb.2018.01118)
Supplement: Supplementary file 3 [file Data_Sheet_3.pdf]

Supplementary Material

Genetic determinants associated with *in vivo* survival of *Burkholderia cenocepacia* in the *Caenorhabditis elegans* model

Yee-Chin Wong[1], Moataz Abd El Ghany[2,3], Raeece Naeem[2], Soon-Joo Yap[4], Chee-Choong Hoh[4], Arnab Pain[2], Sheila Nathan[1,*]

*** Correspondence:** Sheila Nathan: sheila@ukm.edu.my

1 Supplementary Figures and Tables

1.1 Supplementary Figures

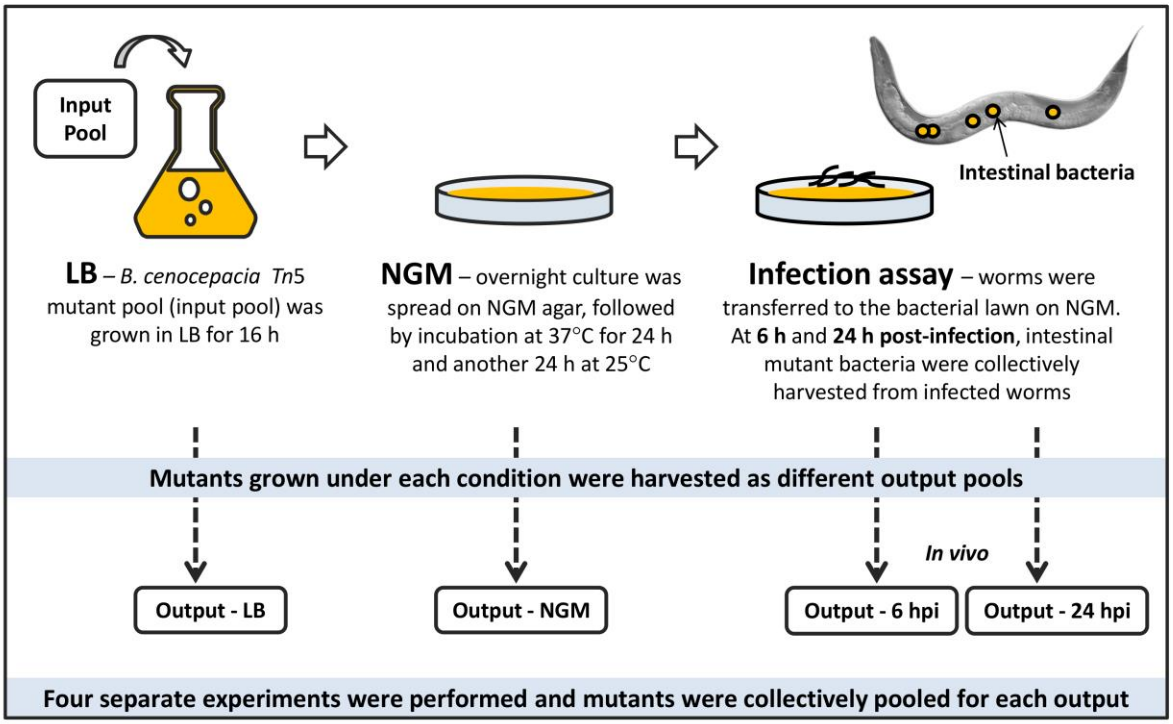

Figure 1. Schematic diagram of harvesting mutants for each output pool. Transposon mutants (approximately 1 x 10^{10} CFU) from the input pool were grown in 50 mL LB broth at 37°C. A 100 µL aliquot of the overnight culture was spread evenly on NGM agar and an additional 5 mL of the culture was kept as **output-LB**. The NGM plates were incubated at 37°C for 24 h and allowed to equilibrate to room temperature for 24 h. The bacterial lawn was washed and collected as **output-NGM**. To establish large-scale infection, thousands of young adult worms were transferred directly onto the NGM agar pre-seeded with input pool mutants. At 6 and 24 h post-infection (hpi) mutants were collected from worm intestines as *in vivo* output pools

(**output-6 hpi** and **output-24 hpi**) (see Materials and Methods). Four experiments were performed and mutants obtained from all experiments were collectively pooled as the final output pools for TraDIS.

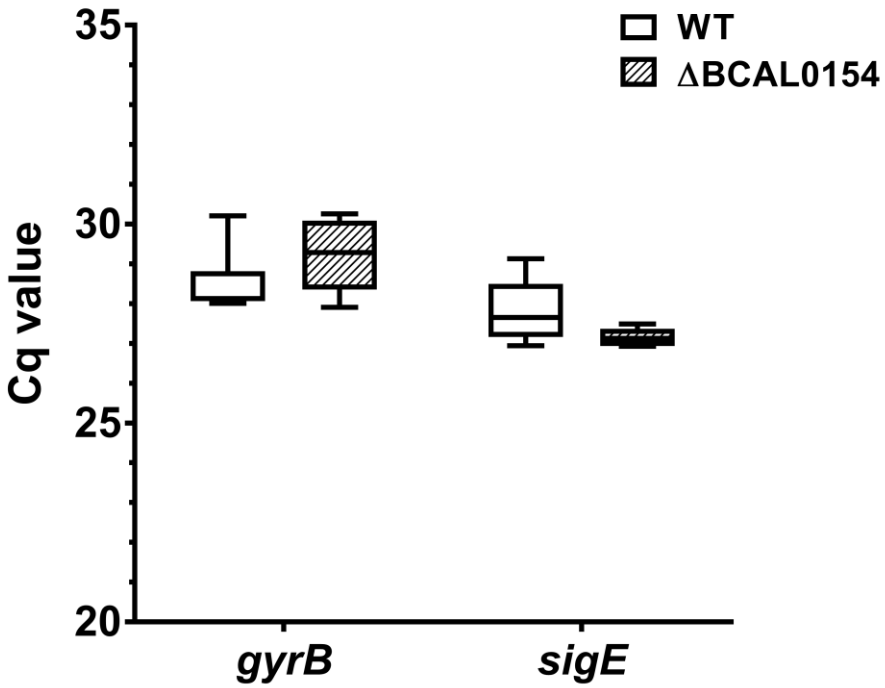

Figure 2. Expression levels of two selected reference genes *gyrB* and *sigE* in *B. cenocepacia* J2315 wild type (WT) and ΔBCAL0154 mutant. The median value and the minimum and maximum Cq were calculated using two biological replicates of both tested samples (WT and ΔBCAL0154) and three technical replicates of each biological replicate. Statistical analysis was performed using unpaired, two-tailed Student's *t*-test and no significant difference was observed between WT and the ΔBCAL0154 mutant for both *gyrB* and *sigE* ($p > 0.05$).

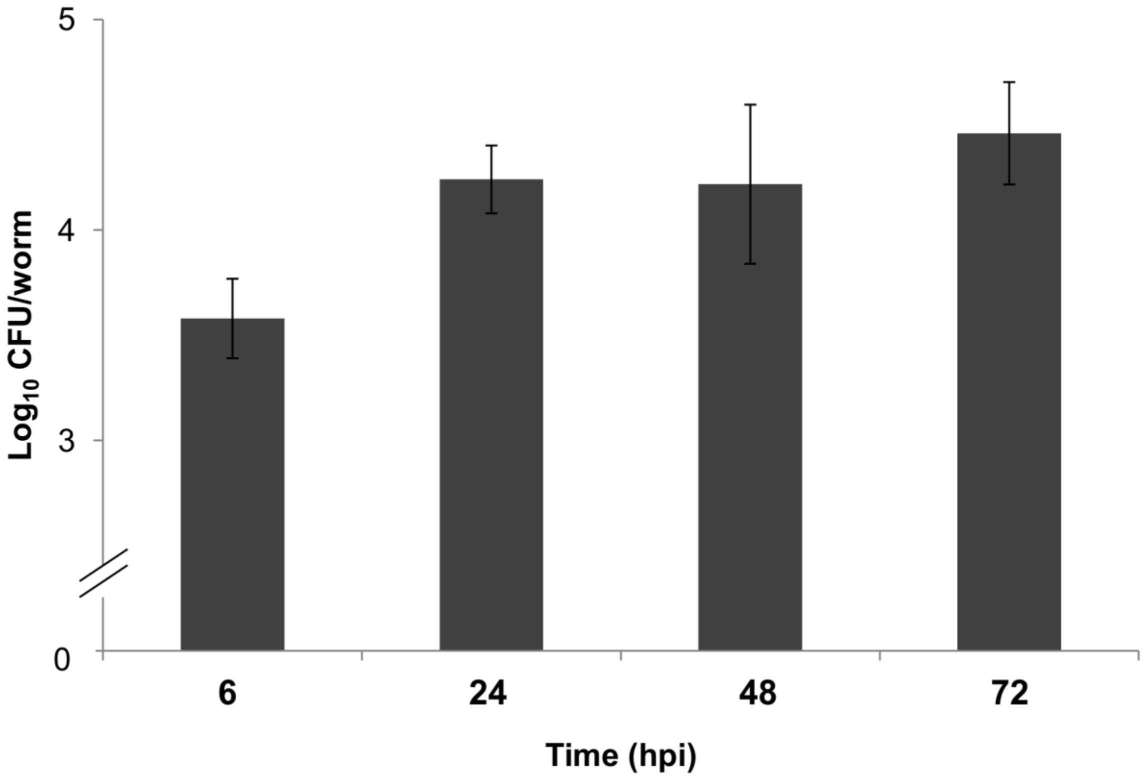

Figure 3. Colonization of *B. cenocepacia* in the *C. elegans* intestinal lumen. The number of *B. cenocepacia* J2315 (wild type) colonizing the *C. elegans* intestinal lumen at 6 h, 24 h, 48 h and 72 h post-infection (hpi). Data are mean bacterial CFU (log_{10}) per worm ± SD of three replicates; for each replicate, bacteria were enumerated from 10 infected worms. At early infection (6 hpi), the number of intestinal bacteria residing in a single worm is ~10^3 CFU. At 24 h post-infection, the number of intestinal bacteria increased and remained constant up to 72 h post-infection i.e. ~10^4 CFU per worm.

frontiers

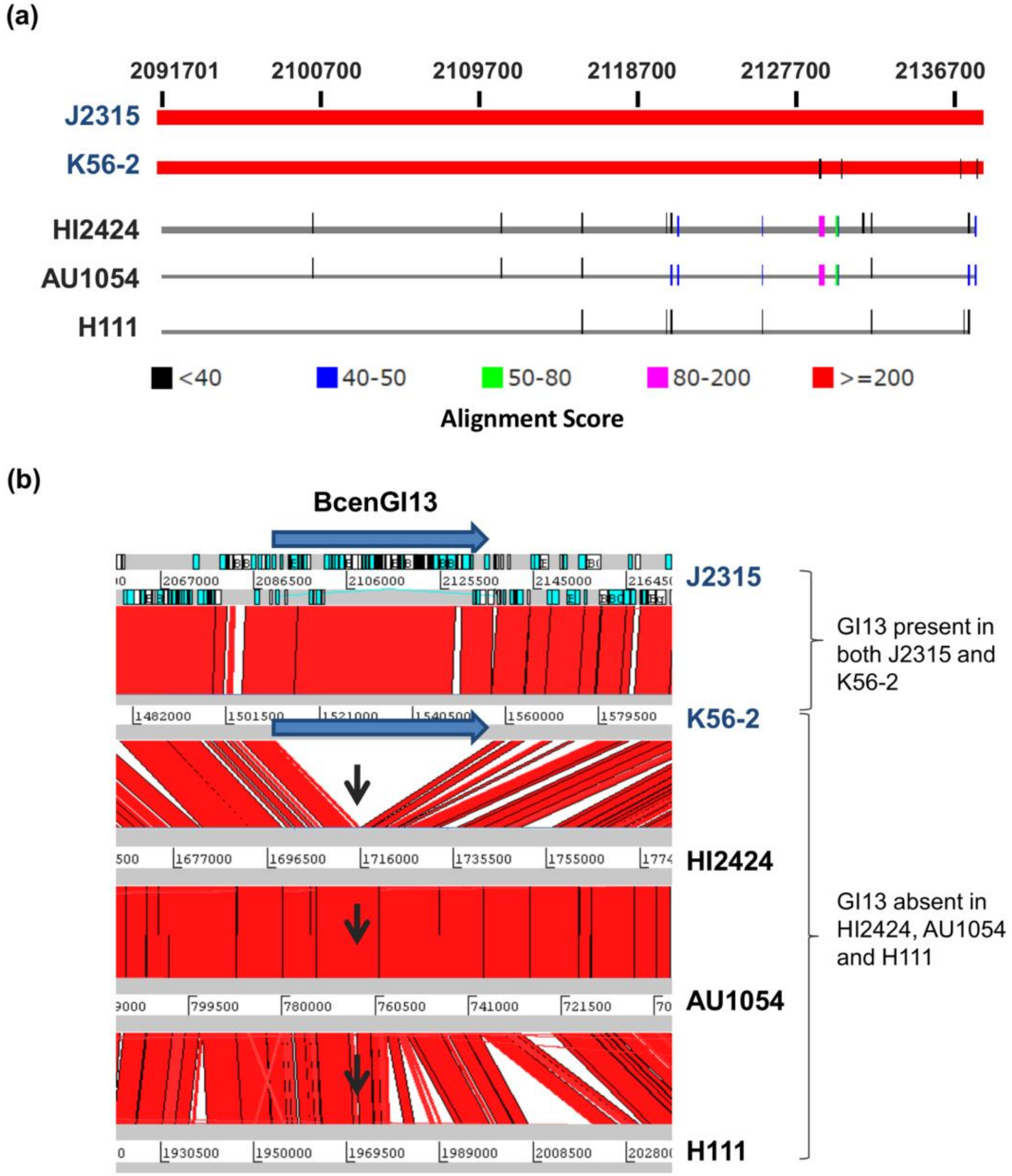

Figure 4. Graphic representations of **(A)** Blast results and **(B)** Artemis Comparison Tool (ACT) analysis of *B. cenocepacia* J2315 genomic island BcenGI13 against genomes of selected *B. cenocepacia* strains (K56-2, HI2424, AU1054 and H111). As shown, BcenGI13 is present in J2315 and K56-2 which belong to the epidemic ET12 lineage but are absent in the other strains (AU1054, HI2424 and H111).

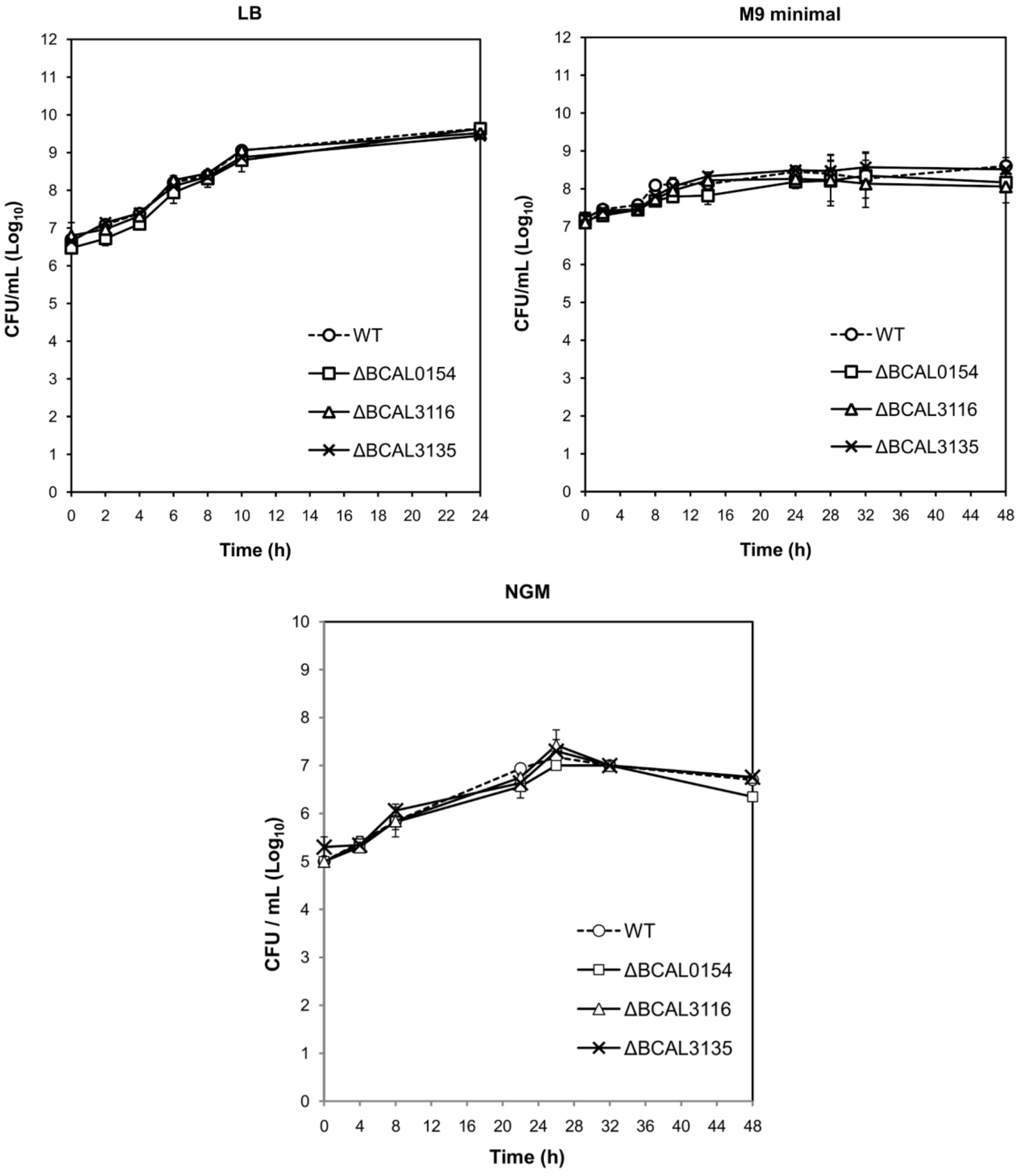

Figure 5. *In vitro* growth of *B. cenocepacia* J2315 wild type (WT) and mutants in LB, M9 minimal media and liquid NGM. Data are presented as mean ± standard error of the mean (SEM) from two independent assays. When compared to wild type, no difference in growth rate was observed for all mutants in all media (unpaired, two-tailed Student's t-test; $p > 0.05$).

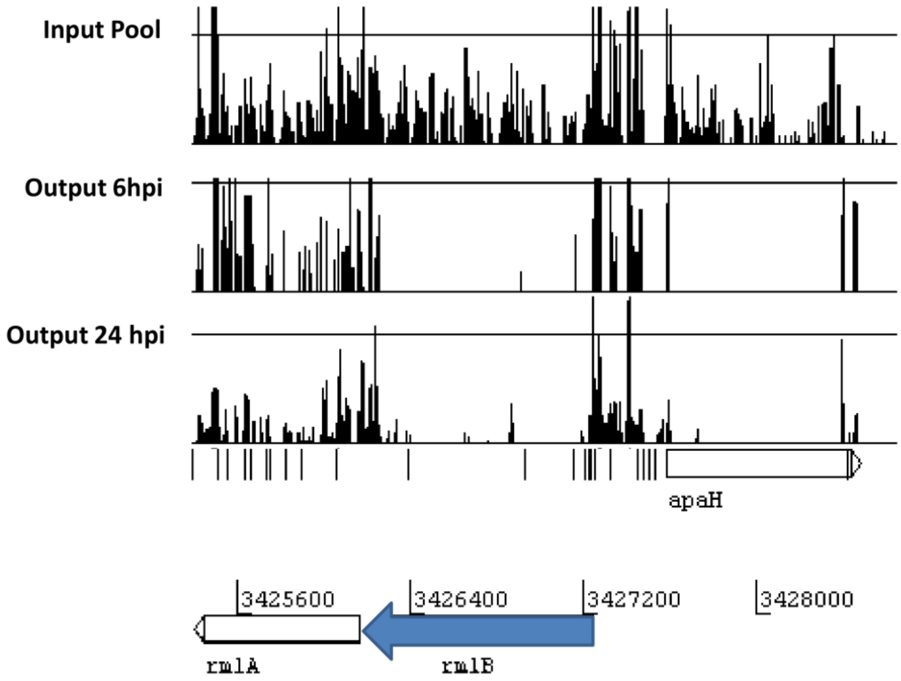

Figure 6. Insertion profile of *rmlB* (BCAL3135) taken from the initial input pool, output-6 hpi and output-24 hpi. Each vertical line represents one unique TIS with the height corresponding to the sequencing depth of the unique read. *rmlB* (BCAL3135), represented by blue arrow, was predicted as non-essential in our TraDIS analysis on input pool, as disruption of *rmlB* by transposon insertions does not affect the viability of the mutant. In both output pools (6 and 24 hpi), the number of reads for each insertion was significantly reduced (log_2 fold change < -2; $p < 0.05$), suggesting a decrease in relative mutant abundance after *in vivo* selection.

1.2 Supplementary Tables

Table 1: Oligo/primers used in this study

Oligo/primer	Sequence (5' - 3')	Description
**TraDIS**		
MP_ Ad_a	GTGACTGGAGTTCAGACGTGTGCTCTTCCGATC*T	Adapter [a]
MP_ Ad_b	p-GATCGGAAGAGCGTCGTGTAGGGAAAGAGTG-amino	
Tra_Fp	AATGATACGGCGACCACCGAGATCTACACCTGATCTAGA GTCGACCTGCAGGCATGCAAGCTTCAG	Amplification of TraDIS library; Forward and reverse primer[b](1)
Tra_Mp_Rp	CAAGCAGAAGACGGCATACGAGATNNNNNNNGTGACTGG AGTTCAGACGTGT	
qPCR_P5	AATGATACGGCGACCACCGA	TraDIS library quantification by qPCR
qPCR_P7	CAAGCAGAAGACGGCATACGA	
Tra_SeqP	AGGCATGCAAGCTTCAGGGTTGAGATGTGTA	Sequencing primer
Tra_IndP	GATCGGAAGAGCACACGTCTGAACTCCAGTCAC	Index primer[c]
**Mutant Construction**		
**BCAL0154**		
BCAL0154_US _F	TTTCGCAAAACTGTCGTCCG	Gene BCAL0154, upstream fragment, product size: 335 bp
BCAL0154_US_R	GCCAGCAGGTCCTTGTAAGA	
BCAL0154_DS_F	TCTTACAAGGACCTGCTGGCGCAAGAACCGCGAACAGTTT	Gene BCAL0154, downstream fragment, product size: 336 bp
BCAL0154_DS_R	CGCAACTGCGTGCTTATCTG	
**BCAL3116**		
BCAL3116_US_F	GTTCGGCAATTTCGACGCTT	Gene BCAL3116, upstream fragment, product size: 332 bp
BCAL3116_US_R	TCGGCTTGATCGTGTAATGGA	
BCAL3116_DS_F	TCCATTACACGATCAAGCCGACGCACGCTGATGGAAGCA T	Gene BCAL3116, downstream fragment, product size: 270 bp
BCAL3116_DS_R	ACCGGTTAACGAGTGGATGG	
**BCAL3135**		
BCAL3135_US_F	GTGTTTCGGCTGCGTAGGAA	Gene BCAL3135, upstream fragment, product size: 343 bp
BCAL3135_US_R	CCAGGATCATGAGGCTCCCT	
BCAL3135_DS_F	AGGGAGCCTCATGATCCTGGAGACGGTCGACTGGTATCTC	Gene BCAL3135, downstream fragment, product size: 345 bp
BCAL3135_DS_R	TGGATGTTCATCCCCCACTG	

**OriT of pEXKm5**			
OriT_F	TCCGCTGCATAACCCTGCTTC		Validation step to confirm the absence of oriT Product size: 236
OriT_R	CAGCCTCGCAGAGCAGGATTC		

Oligo/primer	Sequence (5' - 3')	Product size	References
**qRT-PCR**			
BCAL0421_GyrB_F	GTTCCACTGCATCGCGACTT	109	Peeters, E et al. 2010
BCAL0421_GyrB_R	GGGCTTCGTCGAATTCATCA		
BCAM0918_SigE_F	AGGAAACCAACCGTCAGATG	194	O'Grady et al. 2009
BCAM0918_SigE_R	GCGACGGTATTCGAACTTGT		
BCAL0114_FliC_F	GCGTGTCGATGATTCAAACGGCAT	159	O'Grady et al. 2009
BCAL0114_FliC_R	TCACTTCCTGGATCTGCTGCGAAA		
BCAL0524_FliG_F	GTATTCAAGTTCCTCGCGCC	163	This study
BCAL0524_FliG_R	AGCGGATGTACTCGCTTGAA		
BCAL0124_FlhD_F	ATGAGCGCTACCAGCGAAAT	107	This study
BCAL0124_FlhD_R	CGGAACATACCCATCGCCTT		
BCAL1525_Flp_F	GTCGAGTACGGACTCATCGC	104	This study
BCAL1525_Flp_R	TGGTTCGCGATGTAGGTGAA		
BCAL0134_CheB1_F	CAACAACCCGCTCGTCAGTA	155	This study
BCAL0134_CheB1_R	CGAACGATTTCGTGAAGCCC		

[a]Both oligonucleotides were modified from Illumina adapters. Asterisk indicates phosphorodioate bond, p represents 5' phosphate modifier and amino represents 3' amino modifier. Underlined is a 12-bp complementary region, both oligonucleotides anneal to form a Y-shaped adapter.

[b]N signifies 7 bp barcodes as described by Meyer and Kircher (2010).

[c]Index primer was designed according to Illumina primer sequences.

Oligonucleotide sequences © 2007-2012 Illumina, Inc. All rights reserved. Derivative works created by Illumina customers are authorized for use with Illumina instruments and products only. All other uses are strictly prohibited.

TraDIS library preparation: PCR Cycle Conditions

Template: adapter-ligated DNA fragments (200 ng)

Primers: Tra_Fp and Tra_MP_Rp (each sample uses reverse primer with different index or barcode)

94°C	2 min	
94°C	30 sec	22 cycles
65°C	30 sec	
72°C	30 sec	
72°C	5 min	

Table 2: Summary of TraDIS results from 4 independent sequencing libraries and the respective number of transposon insertions detected in each output pool

**Output pools**	**Total reads**	**No. of reads with transposon tags (%)**	**No. of reads mapped to the reference genome (%)**	**No. of unique insertion sites**
in vitro				
**LB**	31,119,700	30,130,787 (96.8)	25,139,969 (83.4)	**535,285**
**NGM**	32,502,780	31,507,242 (96.9)	26,603,280 (84.4)	**495,747**
in vivo				
**6 hpi**	26,321,418	25,708,653 (97.7)	19,797,123 (77.0)	**128,552**
**24 hpi**	25,225,930	24,412,174 (96.8)	16,632,298 (68.1)	**323,871**

Table 3: Summary of results obtained from phenotypic characterization experiments conducted for all three deletion mutants ΔBCAL0154, ΔBCAL3116 and ΔBCAL3135 compared to wild type strain J2315 (WT).

Strains	*In vitro* growth (doubling time; hours)		Swimming (diameter; cm)		Swarming (diameter; cm)		Biofilm formation		Killing assay (mean time to death; hours ± SD)
	LB	M9	25°C (72 h)	37°C (48 h)	25°C (120 h)	37°C (48 h)	25°C	37°C	
**Wild type**	1.19 ± 0.05	3.67 ± 0.30	4.35 ± 0.52	6.84 ± 0.59	3.08 ± 0.15	3.92 ± 0.15	-	-	147.5 ± 4.5
**ΔBCAL0154**	1.02 ± 0.15	4.17 ± 0.69	0.8 ± 0.13 <	2.75 ± 0.22 <	1.7 ± 0.03 <	2.37 ± 0.13 <	> ****	> ****	223.6 ± 4.4 ****
**ΔBCAL3116**	1.08 ± 0.15	4.10 ± 0.53	2.06 ± 0.49 <	5.7 ± 0.40 <	1.25 ± 0.05 <	3.8 ± 0.13 NS	> **	> ****	178.1 ± 6.2 ****
**ΔBCAL3135**	1.25 ± 0.11	3.39 ± 0.19	4.18 ± 0.58 NS	7.45 ± 0.75 NS	3.1 ± 0.13 NS	4.12 ± 0.02 NS	NS	NS	183.2 ± 5.1 ****

For *in vitro* growth analysis and motility assays, data are presented as mean ± SEM obtained from two independent experiments at different incubation times. For motility and biofilm assays, < indicates "decreased" and > indicates "increased" values compared to wild type, whereas NS indicates "no significant difference". The asterisks represent *P* values of the WT versus mutants (Unpaired, two-tailed Student's *t*-test; $**p < 0.01$, $****p < 0.0001$). For the killing assay, mean times-to-death (TD_{mean}) were determined using the Kaplan-Meier nonparametric survival analysis. The asterisks represent *P* values of the WT TD_{mean} versus mutants ($****p < 0.0001$).

2 References

Meyer, M., and Kircher, M. (2010). Illumina sequencing library preparation for highly multiplexed target capture and sequencing. *Cold Spring Harb. Protoc.* 2010. doi:10.1101/pdb.prot5448

O'Grady, E. P., Viteri, D. F., Malott, R. J., and Sokol, P. A. (2009). Reciprocal regulation by the CepIR and CciIR quorum sensing systems in *Burkholderia cenocepacia*. *BMC Genomics* 10, 441.

Peeters, E., Sass, A., Mahenthiralingam, E., Nelis, H., and Coenye, T. (2010). Transcriptional response of *Burkholderia cenocepacia* J2315 sessile cells to treatments with high doses of hydrogen peroxide and sodium hypochlorite. *BMC Genomics* 11, 90. doi: 10.1186/1471-2164-11-90
